# Co-designing a study on a health interventions with Myanmar migrant workers to address work-related musculoskeletal disorders in Thailand’s seafood industry

**DOI:** 10.1186/s40900-026-00891-8

**Published:** 2026-04-28

**Authors:** Phyu Hnin Hlaing, Phaik Yeong Cheah, Soe Thandar Myint, Smith Boonchutima

**Affiliations:** 1 Faculty of Communication Arts, Chulalongkorn University, Phayathai Road, Pathumwan, Bangkok, 10330 Thailand; 2 Mahidol-Oxford Tropical Medicine Research Unit, Faculty of Tropical Medicine, Mahidol University, 420/6 Rajvithi Road, Tungphyathai, Bangkok, 10400 Thailand; 3 Centre for Tropical Medicine and Global Health, Nuffield Department of Medicine, University of Oxford, Wellington Square, Oxford, OX1 2JD UK; 4 Faculty of Public Health, Mahidol University, 420/1 Ratchawithi Road, Thung Phaya Thai, Ratchathewi, Bangkok, 10400 Thailand; 5 Center of Excellence in Communication Innovation for Development of Quality of Life and Sustainability, Chulalongkorn University, Phayathai Road, Pathumwan, Bangkok, 10330 Thailand

**Keywords:** Patient and public involvement, Study co-design, Community involvement, Migrant health, Occupational health, Musculoskeletal disorders, Digital health intervention, Intervention development, Thailand, Myanmar workers, Participatory research

## Abstract

**Background:**

Myanmar migrant workers in Thailand’s seafood processing industry develop work-related musculoskeletal disorders from tasks such as repetitive shrimp peeling and standing for extended periods. These workers remain largely unreached by standard health interventions due to language barriers and cultural isolation. Developing effective study for this vulnerable population requires meaningful involvement of workers in the study design process, recognizing their experiential expertise while acknowledging the distinct roles of researchers and community members. This paper documents how involvement with stakeholders such as migrant workers themselves shaped the co-design of a study testing a culturally appropriate digital health intervention for managing existing symptoms and preventing progression of musculoskeletal disorders.

**Methods:**

This was a mixed-methods co-design study combining qualitative community engagement with quantitative expert validation. We conducted engagement sessions with 29 Myanmar migrant workers, organized into three groups by length of work experience (less than 2 years, 2–5 years, more than 5 years). We engaged 5 workplace stakeholders (human resource managers and production line supervisors) and consulted 4 international physical therapy experts for validation. Sessions were structured as conversations. Workers provided substantial input on intervention content, delivery methods, and practical requirements for the planned study. We accommodated their 12-hour work schedules, communicated in Myanmar language, and valued their experiential knowledge as essential for culturally appropriate study design. Thematic analysis identified key themes from worker input. Expert validators assessed the co-designed intervention using Content Validity Index (CVI) methodology.

**Results:**

All participating workers reported hand symptoms including numbness, tingling, and pain. Workers expressed preferences for health information through platforms they used daily, with Facebook emerging as the clear preference over text-based materials or in-person workshops on their only day off. These insights shaped the study intervention: a 4-week intervention with 12 progressive exercises addressing observed strain patterns: forward-leaning postures and repetitive hand movements. Workers specified practical requirements such as exercises must function in small dormitory spaces, outside work hours, without equipment. The peer challenge format emerged from their suggestions about sustained engagement. The co-designed study intervention achieved strong content validity scores from expert validators (I-CVI: 0.95-1.00; S-CVI/Ave: 0.94). The engagement process also highlighted ethical considerations when working with vulnerable migrant populations, including managing power differentials and protecting workers from potential workplace repercussions.

**Conclusions:**

Workers possess essential knowledge about their needs and what works in their circumstances. We found that when research incorporates the input of the intended beneficiaries in study design, the result is a study intervention that is both clinically appropriate and practically usable. The co-designed intervention is now used for evaluation in the implementation research. This paper contributes a documented methodological approach to community involvement with a vulnerable migrant population, demonstrating that structured engagement can produce interventions meeting both worker feasibility requirements and clinical validity standards, while generating an honest account of the ethical tensions such work entails.

## Abstract (plain language summary)

**What is the problem?:**

Myanmar migrant workers in Thailand’s seafood industry perform repetitive tasks like shrimp peeling while standing for long hours. This leads to pain in their hands, necks, backs, and other body parts. Language barriers, limited healthcare access, and demanding work schedules make it difficult for these workers to get help or learn how to prevent these problems.

**What did we do?:**

We worked directly with 29 Myanmar workers, workplace managers, and physical therapy experts to design a study that would test a health program on addressing pain in their hands, necks, backs, and other body parts specifically for this community. We sought input from workers to help shape the study based on what would actually work in their daily lives. We asked about their pain, their access to healthcare, and how they prefer to receive health information.

**What did we find?:**

Every worker we spoke with experienced hand symptoms, and many also had neck, back, or leg pain. Workers told us they prefer getting health information through Facebook videos they can watch during their limited free time, rather than attending workshops on their only day off. They wanted short, practical exercises they could do in their dormitories without special equipment. We also encountered some ethical issues, including ensuring workers felt safe to speak honestly without fear of workplace consequences and addressing the power imbalance between researchers and workers in the co-design process.

**What does this mean?:**

By involving stakeholders including migrant workers themselves in designing this study, we created a Facebook-based program with simple exercises and health tips tailored to their needs that is now ready to be tested in a future study. We describe how migrant workers can be effective contributors in developing study that address their unique challenges, and digital platforms offer a practical way to reach people with demanding work schedules.

## Background

The transformation of Thailand’s seafood industry over recent decades has created one of the world’s largest concentrations of migrant workers in a single industrial sector [1]. More than 300,000 Myanmar nationals now contribute to Thailand’s seafood processing operations, their labor forming the backbone of an industry that generates billions of dollars in export revenue annually [2]. Yet this economic success has come at considerable cost to worker health, including in the form of work-related musculoskeletal disorders (WMSD) that now affect the vast majority of these workers [3, 4].

WMSD represent injuries or disorders of muscles, tendons, joints, and supporting structures caused or aggravated by workplace activities and ergonomic stressors [5]. In seafood processing environments, these conditions arise from the repetitive nature of tasks such as shrimp peeling, prolonged standing during fish preparation, and the sustained awkward postures required for production line work [6]. The cold, wet conditions typical of seafood processing compound these physical stresses, creating an occupational environment that systematically damages worker health over time [4].

Recent epidemiological studies have documented work-related musculoskeletal disorder prevalence rates of 65 to 85% among Myanmar migrant workers in Thai seafood facilities, figures that exceed those found in most other industrial settings [3, 7]. These conditions impose direct costs through medical treatment and lost of productivity, while creating broader social costs as workers struggle with chronic pain and reduced quality of life [8]. The economic pressures that drive migration create a cycle in which immediate survival needs override long-term health considerations [9].

Traditional occupational health interventions have consistently failed to reach this population effectively [10]. Language barriers represent only the most obvious challenge. Cultural differences in health beliefs, social hierarchies within migrant communities, legal status concerns, and limited access to healthcare services all contribute to the ineffectiveness of conventional health promotion strategies [9, 11]. Myanmar migrant workers often remain isolated from mainstream health communication efforts, creating a population at high risk with limited access to preventive interventions [12].

Patient and Public Involvement and Engagement (PPIE) approaches offer a fundamentally different paradigm for health research development [13]. These methods work with communities to identify needs, preferences, and potential solutions [14]. This approach has gained recognition as essential for developing research that are not merely clinically sound but ethical, genuinely acceptable and effective for the communities for whom the solutions are meant for [15]. For marginalized communities such as migrant workers, involvement approaches are particularly important as they can help addresses power imbalances inherent in traditional research relationships and recognize community members as possessing valuable experiential knowledge [16].

However, implementing PPIE approaches presents well-documented ethical and practical challenges. Scholars have noted the risk of tokenistic involvement where community input is sought but does not meaningfully influence outcomes [17]. Power differentials between between researchers and community members persist even in participatory frameworks, and genuinely sharing decision-making authority requires ongoing negotiation rather than procedural solutions [18]. These challenges are particularly acute when working with populations who may feel constrained in expressing disagreement with researcher proposals. Our approach attempted to address these concerns while acknowledging that perfect power-sharing may be an aspirational rather than achievable goal.

Our goal was to co-design a study on health interventions with relevant stakeholders to prevent work-related musculoskeletal disorders in Thailand’s seafood industry [19].

This paper emerged from recognition that existing approaches to occupational health among Myanmar migrant workers were failing to address a serious and growing health crisis [3, 20]. We sought to document a community involvement approach to study development that would incorporate worker input throughout the design process [13]. To our knowledge, no published study has documented a structured community involvement process for developing a digital health intervention with Myanmar migrant workers in Thailand’s seafood industry. This paper addresses that gap. Our work contributes both a validated, culturally appropriate intervention ready for testing in future research and a methodological framework for engagement with vulnerable communities that may be applied to similar populations globally [15, 21].

The co-design process, Phase I of our project, took place in 2024, involving extensive engagement with migrant workers, management personnel, and health experts. Through this process, we aimed to understand not merely what interventions might be theoretically beneficial, but what approaches would genuinely resonate with workers facing daily economic and physical pressures. This paper documents Phase I of the project, which describes the engagement methodology, examines how worker input shaped study design, and presents the validated interventions as an outcome of collaboration that will be evaluated in subsequent implementation research (Phase II) [19].

## Methods

### Design and theoretical framework

These activities employed a co-design approach grounded in Patient and Public Involvement and Engagement (PPIE) methodology to develop a cultural tailored intervention for preventing work-related musculoskeletal disorders among Myanmar migrant workers. Reporting follows the GRIPP2 guidelines for patient and public involvement in research [14]. The theoretical foundation drew from community-based participatory research principles [22]; the Health Belief Model [23] and cultural adaptation framework [24].

The Health Belief Model informed the framing of educational content around perceived susceptibility to injury and barriers to preventive action; the cultural adaptation framework guided language choices, visual imagery, and the structuring of peer challenges around community participation rather than individual competition. Worker-specified requirements were documented and treated as design constraints rather than preferences to be weighed against researcher judgment: when expert recommendations diverged from what workers identified as feasible, worker feasibility took precedence. We acknowledge tension between participatory framing and the traditional qualitative methods. While PPIE principles emphasize shared power, practical constraints required researcher-designed procedures and analysis. We addressed this by structuring sessions as open conversations, allowing worker priorities to shape discussion direction. Our approach represents community involvement rather than full partnership with equal decision-making authority.

Phase I outputs include documented co-design methodology, validated intervention content, implementation protocols, and procedures for Phase II effectiveness study.

### Setting and context

The majority of our activities took place at a seafood processing facility located in Samut Sakhon Province, Thailand, employing approximately 900 Myanmar migrant workers. Workers perform repetitive tasks including shrimp peeling and 12-hour shifts in cold, wet conditions, creating systematic exposure to musculoskeletal disorder risk factors [3, 5]. Workers live in adjacent company dormitories, forming a contained community with strong social networks.

### Co-design process

The co-design process involved three parallel engagement activities conducted between March and May 2024, each contributing distinct knowledge to our project.

#### Engagement with migrant workers

##### Participants

Twenty-nine Myanmar migrant workers stratified by level of experience on the job: fewer than 2 years (*n* = 10), 2–5 years (*n* = 11), and more than 5 years (*n* = 8). Ages ranged from 22 to 45 years (mean 32.4); 62% women. All were actively employed in seafood processing roles. Recruitment through workplace announcements yielded convenience sampling. Participants completed brief demographic forms in Burmese prior to engagement sessions (Table 1).

**Table 1:** Members demographics and work characteristics

Characteristic	Years of working in the seafood industry			
	Group 1 (< 2 years)	Group 2 (2–5 years)	Group 3 (> 5 years)	Total Sample
Sample size	10	11	8	29
Age (years)				
Mean (SD)	28.2 (4.1)	33.1 (5.8)	37.8 (6.2)	32.4 (6.3)
Range	22–35	25–42	28–45	22–45
Gender, n (%)				
Female	7 (70.0)	7 (63.6)	4 (50.0)	18 (62.1)
Male	3 (30.0)	4 (36.4)	4 (50.0)	11 (37.9)
Work experience				
Mean months (SD)	8.4 (6.2)	36.8 (12.1)	78.6 (38.4)	37.2 (32.8)
Range (months)	2–18	24–58	60–156	2-156
Primary work role, n (%)				
Shrimp peeling	8 (80.0)	7 (63.6)	4 (50.0)	19 (65.5)
Fish cutting/preparation	1 (10.0)	3 (27.3)	3 (37.5)	7 (24.1)
Quality control	1 (10.0)	1 (9.1)	1 (12.5)	3 (10.3)
Shift pattern, n (%)				
Day shift (6am-6pm)	6 (60.0)	7 (63.6)	5 (62.5)	18 (62.1)
Night shift (6pm-6am)	4 (40.0)	4 (36.4)	3 (37.5)	11 (37.9)
Housing arrangement, n (%)				
Company dormitory	10 (100.0)	11 (100.0)	6 (75.0)	27 (93.1)
Private rental	0 (0.0)	0 (0.0)	2 (25.0)	2 (6.9)
Facebook usage, n (%)				
Daily	9 (90.0)	10 (90.9)	7 (87.5)	26 (89.7)
Weekly	1 (10.0)	1 (9.1)	1 (12.5)	3 (10.3)
Previous workplace injury, n (%)				
Yes	2 (20.0)	5 (45.5)	6 (75.0)	13 (44.8)
No	8 (80.0)	6 (54.5)	2 (25.0)	16 (55.2)

Note: These demographic characteristics describe the 29 Myanmar migrant workers who served as co-design partners in intervention development. This sample provided diverse perspectives across work experience levels, gender, and job roles, informing comprehensive understanding of occupation health needs and intervention design requirements

##### Engagement session

Three group sessions were conducted on March 17, 2024 (Sunday, worker’s only day off), each lasing approximately 60 min. Groups comprised 8–11 workers stratified by experience level to facilitate peer-level discussion. The principal investigator (PHH) led sessions in Burmese with a Myanmar medical doctor as note-taker. All sessions were audio-recorded following written informed consent.

##### Topics explored

Current understanding of work-related musculoskeletal disorders, personal symptom experiences and their daily impact, cultural factors affecting health intervention acceptance, existing strategies for managing pain (including frequency of over-the-counter medication use, barriers to accessing healthcare, preferences for intervention delivery methods (timing, format, digital vs. in-person), receptivity to gamification elements (challenges, peer comparison, rewards), and practical constraints determining intervention feasibility within their work schedules and living situations.

##### Key contributions

Workers provided universal hand symptom as the priority concern, described healthcare barriers (language difficulties, discrimination, financial constraints), revealed weekly over-the-counter medications purchases as primary pain management, expressed preferences for Facebook delivery over workshops on their day off, specificd practical requirements (dormitory-performable, no equipment, 15–20 min maximum), and endorsed peer challenges emphasizing participation over performance.

#### Engagement with workplace stakeholders

##### Participants

Five stakeholders including one Thai human resources manager, two Burmese line managers, and two Thai line managers, all with more than 5 years experience in roles involving worker health and safety.

##### Engagement session

Individual interviews lasting 40–60 min, conducted between March and April 2024 in participants’ preferred language (Thai or Burmese). Interviews were led by PHH (for Burmese-speaking participants) and SB (for Thai-speaking participants).

##### Topics explored

Organizational practices affecting worker health, workplace culture and communication patterns, observed musculoskeletal challenges among workers, existing health communication strategies, feasibility of implementing workplace-based interventions, and organizational readiness for supporting research activities.

##### Key contributions

Stakeholders confirmed daily hand pain complaints across the workforce, identified structural constraints (12-hour shifts, limited days off) that would restrict workers’ capacity for time-intensive interventions, clarified organizational health service policies (workplace insurance covers only acute injuries, not chronic conditions), and validated dormitory-based delivery as feasible within existing organizational structures while noting workers need to participate outside work hours.

#### Engagement with clinical experts

##### Participants

Four experts (two Myanmar physical therapists, two Singapore-based physical therapists/exercise physiologists), all with more than 2 years specialized experience in musculoskeletal health and occupational therapy.

##### Methods

Expert reviews combining 90-minute video consultations with written evaluation using standardized Content Validity Index (CVI) instruments. Experts were selected on the basis of a minimum of two years’ specialised experience in musculoskeletal health and occupational therapy, with regional experience in Southeast Asian contexts prioritised. Item-level CVI (I-CVI) scores of 0.78 or above were considered acceptable, a scale-level average CVI (S-CVI/Ave) of 0.90 or above was considered indicative of excellent content validity, both following Polit and Beck (2006) [25]. During consultations, experts reviewed the proposed intervention components (exercise selections, delivery format, cultural adaptation approaches, and assessment tools). Following consultations, experts independently rated each intervention component on a 5-point scale (1 = strongly disagree to 5 = strongly agree) across four dimensions: relevance, clarity, cultural appropriateness, and clinical soundness. Expert consultations occurred from 14th to 25th March, after initial worker engagement sessions documented worker preferences.

##### Topics reviewed

Exercise selection rationale addressing upper and lower crossed syndromes, progression strategies, cultural adaptation approaches, safety protocols, implementation feasibility within typical constraints, and assessment form validity.

##### Key contributions

Experts validated exercise selections as appropriate for documented occupational exposures, confirmed cultural adaptation approaches as suitable for target population, provided safety guidance and contraindications, and assessed intervention components on relevance, clarity, cultural appropriateness for specific exercises, and provided quantitative rating across all intervention components that informed CVI calculations.

### Intervention development process

Exercise selection addressed worker-reported symptom patterns: universal hand symptoms prioritized hand and wrist exercises; neck pain prevalence informed cervical mobility work; back pain guided postural exercises. While originally designed for prevention, the intervention was adapted to serve dual purposes: managing existing symptoms and preventing further deterioration, given that all participating workers already experienced musculoskeletal pain. The lead researcher (PHH) identified evidence-based exercises addressing these patterns and presented options to workers for feasibility feedback. Workers specified that exercises must be performable in dormitory spaces without equipment, maximum 15–20 min daily. When expert recommendations (longer durations, more repetitions) diverged from worker-specified constraints, we prioritized worker feasibility while working with experts to maintain therapeutic benefit.

The intervention incorporated gamification elements based on evidence that game design features enhance motivation and engagement with health-promoting behaviors [26, 27]. By incorporating elements such as points, rewards, social interaction, and achievement recognition, gamification approaches can sustain participation in health interventions [28], particularly when delivered through familiar digital platforms suited to demanding work schedules [29, 30]. These design principles aligned with worker suggestions about competitive games and peer comparison. More specifically, gamification features reflect the Health Belief Model’s emphasis on cues to action and self-efficacy: points and weekly challenges function as repeated behavioural cues, while the peer challenge format emphasising participation over performance was designed to support self-efficacy among workers with varying degrees of physical limitation. Workers refined proposed features (points, leaderboards, weekly challenges), suggesting recognition emphasize participation over performance to avoid discouraging those with physical limitations.

The final program comprised 12 progressive exercises addressing the upper and lower crossed syndromes: simple mobility exercises (chin tucks, tendon gliding) providing immediate symptom relief; stretching exercises (hip flexors, chest, palm and neck stretches) for restricted movement; and strengthening exercises (glute bridges, planks, prone cobra) for muscle weakness from sustained work postures while preventing further strain. All content was developed in Burmese with cultural imagery and Myanmar values around community support.

### Data analysis

#### Qualitative data analysis

Thematic analysis following established procedures [31]. PHH and STDM coded transcripts independently with regular meetings to discuss emerging themes and resolve disagreements through consensus. Analysis began during engagement, with initial themes explored in subsequent sessions. Major themes were developed through iterative discussion with attention to cultural factors, power dynamics and practical constraints [9, 11].

We acknowledge that stakeholders were not involved in formal coding given their 12-hour shifts and limited personal time. This means our interpretation, while informed by extensive engagement, was researcher-driven. Credibility was supported by independent dual coding between PHH and STDM, with disagreements resolved through consensus discussion. Dependability was maintained through reflective memos kept by PHH throughout the engagement period, recording analytical decisions and their rationale as they were made. Member checking was not conducted, given the constraints on workers’ time; future work with similar populations might incorporate a structured session presenting summary findings to a representative subset of workers for validation, as a practical means of reducing researcher-driven interpretation without placing undue demands on participants’ limited personal time. Direct participant quotations are not reproduced in this paper to protect the confidentiality of workers from a small, identifiable workforce at a single facility.

##### Quantification of worker-reported behaviors

While engagement sessions were primarily qualitative, we documented frequency of specific behaviors reported by workers during discussions. We counted how many of the 29 participants reported weekly medication purchases and daily social media usage when these topics arose during sessions. These counts (reported as percentages) provide context for intervention design decisions while acknowledging they represent self-reported data from discussion rather than validated survey instruments.

#### Quantitative data analysis

Expert review data analyzed using Content Validity Index (CVI) calculations. Item-level indices (I-CVI) calculated for each intervention component. Scale-level indices calculated using average (S-CVI/Ave) and universal agreement (S-CVI/UA) methods, providing quantitative validation that the community-driven achieved clinical appropriateness.

### Ethical considerations

The Research Ethics Review Committee at Chulalongkorn University approved Phase I and II of this study (COA No. 164/67). Following ethical approval, we obtained formal permission from the factory to conduct research activities at their facility. All participants received detailed information in their preferred language with Burmese translations reviewed by native speakers. Workers were informed that participation was voluntary. They could withdraw at any time and that withdrawal would not affect their employment or access to services.

Recruitment happened through general announcements. Supervisors did not refer workers directly. Participants gave consent in private discussions without management presence. Workers had 48 h to consider their decision after receiving information. The team reminded them repeatedly that choosing not to participate would have no workplace consequences.

Modest compensation was provided and it covered time and expenses without creating undue influence. The research team stored all audio recordings on encrypted devices with access limited to research team. Transcripts removed all identifying information. The team coded or deleted names and other personal details.

## Insights

The structured engagement sessions conducted over a six-month project period with 29 Myanmar migrant workers, 5 workplace stakeholders, and 4 clinical experts yielded insights that shaped intervention content, delivery methods, and implementation protocols. This section presents: (1) worker-identified health concerns (2), key factors shaping study design requirements (3), stakeholder perspectives on study feasibility (4), expert validation results, and (5) final co-designed intervention features.

### Worker-identified health concerns

Among the 29 workers who participated in engagement sessions, a purposive convenience sample drawn from a single facility, all reported hand symptoms (numbness, tingling, pain), identifying this as a universal concern within this group requiring immediate attention. Twenty-five workers (86%) described morning hand stiffness affecting daily activities beyond work. Additional widespread concerns included neck pain (79%), lower back pain (72%), and ankle pain (45%). Workers connected symptoms to specific work demands: neck pain from forward head postures during shrimp peeling, back pain from prolonged standing and forward-leaning postures, ankle pain from standing on hard surfaces during 12-hour shifts.

These worker-identified patterns directly informed exercise selection, prioritizing hand/wrist exercises, cervical mobility work, and postural exercises. Workers across all experience levels reported similar symptoms, though newer workers sometimes described more severe problems, which experienced workers attributed to lack of knowledge about protective techniques learned informally over time (Table 2).

**Table 2:** Worker-reported musculoskeletal symptoms informing intervention design priorities

Body Region/Symptom	Group 1 (< 2 years) *n* = 10	Group 2 (2–5 years) *n* = 11	Group 3 (> 5 years) *n* = 8	Total Sample *n* = 29	*p*-value*
Hand/Wrist Symptoms					
Any hand symptoms	10 (100.0)	11 (100.0)	8 (100.0)	29 (100.0)	-
Numbness/tingling	10 (100.0)	11 (100.0)	8 (100.0)	29 (100.0)	-
Morning stiffness	8 (80.0)	9 (81.8)	8 (100.0)	25 (86.2)	0.45
Pain during work	9 (90.0)	10 (90.9)	8 (100.0)	27 (93.1)	0.67
Grip weakness	6 (60.0)	8 (72.7)	7 (87.5)	21 (72.4)	0.32
Neck Symptoms					
Neck pain	7 (70.0)	9 (81.8)	7 (87.5)	23 (79.3)	0.58
Neck stiffness	6 (60.0)	8 (72.7)	6 (75.0)	20 (69.0)	0.71
Headache	5 (50.0)	6 (54.5)	5 (62.5)	16 (55.2)	0.82
Shoulder Symptoms					
Shoulder pain	6 (60.0)	7 (63.6)	6 (75.0)	19 (65.5)	0.74
Shoulder stiffness	5 (50.0)	6 (54.5)	5 (62.5)	16 (55.2)	0.82
Back Symptoms					
Lower back pain	7 (70.0)	8 (72.7)	6 (75.0)	21 (72.4)	0.96
Upper back pain	5 (50.0)	7 (63.6)	5 (62.5)	17 (58.6)	0.73
Back stiffness	6 (60.0)	7 (63.6)	6 (75.0)	19 (65.5)	0.74
Lower Extremity Symptoms					
Ankle pain	4 (40.0)	5 (45.5)	4 (50.0)	13 (44.8)	0.86
Leg cramps	3 (30.0)	4 (36.4)	4 (50.0)	11 (37.9)	0.61
Varicose veins	0 (0.0)	2 (18.2)	3 (37.5)	5 (17.2)	0.03†
Severity Indicators					
Sought medical treatment	2 (20.0)	5 (45.5)	6 (75.0)	13 (44.8)	0.02†
Steroid injection received	0 (0.0)	2 (18.2)	3 (37.5)	5 (17.2)	0.03†
Surgery required	0 (0.0)	1 (9.1)	2 (25.0)	3 (10.3)	0.16
Weekly pain medication use	6 (60.0)	9 (81.8)	8 (100.0)	23 (79.3)	0.08
Work absence due to symptoms	1 (10.0)	3 (27.3)	4 (50.0)	8 (27.6)	0.12

Note: Findings reflect experiences of workers who voluntarily participated and may not represent the broader workforce; however, symptom patterns are consistent with epidemiological research in similar occupational contexts

### Four factors shaping study design requirements

**Fig. 1:**
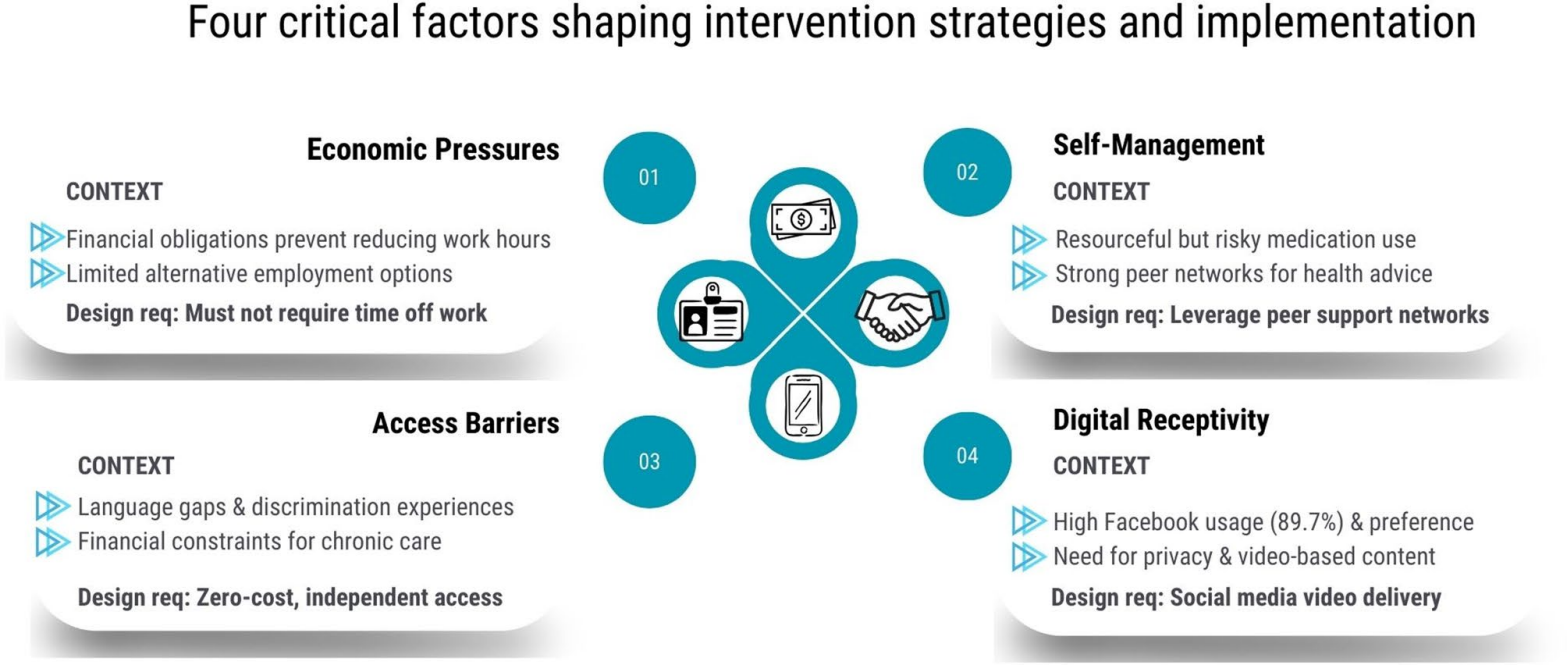
Thematic framework

Engagement sessions revealed four interconnected factors that determined study design and implementation strategies (Fig. 1).

#### Economic pressures shaping health decisions

Workers explained they understood long-term health consequences but could not reduce hours or change jobs given financial obligations to families in Myanmar. This required that exercises be performable outside work hours without reducing earning capacity, participation not require time off work, and content focus on symptom management within existing employment rather than job modifications workers could not implement.

#### Resourceful but risky self-management

Workers demonstrated strong motivation through frequent over-the-counter medication purchases (23 of 29 workers purchasing pain medication at least weekly, 79%), sharing medication information through peer networks, and use of traditional remedies including massage. However, reliance on chronic medication use and limited understanding of injury prevention (some workers continued identical work after surgery) revealed opportunities to provide safer alternatives through exercises and preventive education that acknowledged existing efforts rather than dismissing them.

#### Healthcare access barriers

Workers identified multiple barriers: language difficulties with providers not speaking Burmese, disrespectful interpreters especially in government hospitals, financial stress from paying for chronic conditions through social welfare savings (workplace insurance covered only acute injuries), and discrimination in healthcare settings. These barriers necessitated zero-cost intervention delivered entirely in Burmese through familiar platforms, bypassing healthcare systems where workers felt unwelcome.

#### Receptivity to digital health delivery

Workers expressed strong preferences for Facebook (26 of 29 workers reported daily Facebook use, 89.7%), which offered privacy to access health information without exposing conditions to supervisors, flexible timing suited to demanding schedules, and social features enabling peer support. Workers specifically rejected mandatory workshops on their only day off. They requested video demonstrations over written materials for exercise instruction, and suggested peer engagement would motivate participation, informing incorporation of social recognition and peer challenges.

### Perspectives on study feasibility

Workplace stakeholders confirmed daily hand pain complaints were most frequent supervisory concern. They described workers requiring surgery yet returning to identical conditions, revealing limited organizational injury prevention understanding. Previous successful workshop on lifting techniques demonstrated organizational willingness to support practical, skills-based health education. Looking ahead to Phase II implementation, stakeholders confirmed workforce stability would enable following workers over a 4-week intervention period, though emphasized that all follow-up assessments and participation must occur outside work hours to avoid production disruption.

Stakeholders emphasized resource constraints: approaches requiring significant equipment investment or facility modifications would face barriers. They preferred implementation during existing breaks without affecting production. Organizational constraints aligned well with worker preferences for dormitory-performable exercises without equipment, creating feasibility for both groups. Stakeholders strongly supported dormitory-based delivery, recognizing challenges of workshop attendance given limited worker leisure time.

### Expert review results

Four international physical therapy experts validated the community-driven design through structured Content Validity Index assessment. Quantitative analysis demonstrated excellent agreement: overall S-CVI/Ave reached 0.94 (above 0.90 threshold for excellent validity), with 87% of components receiving acceptable ratings from all experts.

Exercise components received particularly high ratings (I-CVI: 0.95-1.00) for relevance to occupational exposures. Experts appreciated specific focus on upper and lower crossed syndromes as appropriate clinical targets, and progressive structure from mobility to strengthening reflected sound therapeutic principles. Cultural adaptation approaches were validated as appropriate and essential, particularly by the two experts with Myanmar community experience who emphasized importance of language and culturally familiar imagery.

Gamification elements received more varied responses (I-CVI: 0.75 for some components) given limited published evidence, though experts recognized potential for enhanced engagement in communities with limited healthcare access and supported the approach given it emerged from worker suggestions rather than external imposition. Experts provided detailed safety guidance on precautions and contraindications, which were incorporated into final materials [31].

These validation results demonstrated that worker-driven design choices, when combined with clinical expert review, produced an intervention that maintained therapeutic integrity while addressing real-world feasibility constraints.

**Fig. 2:**
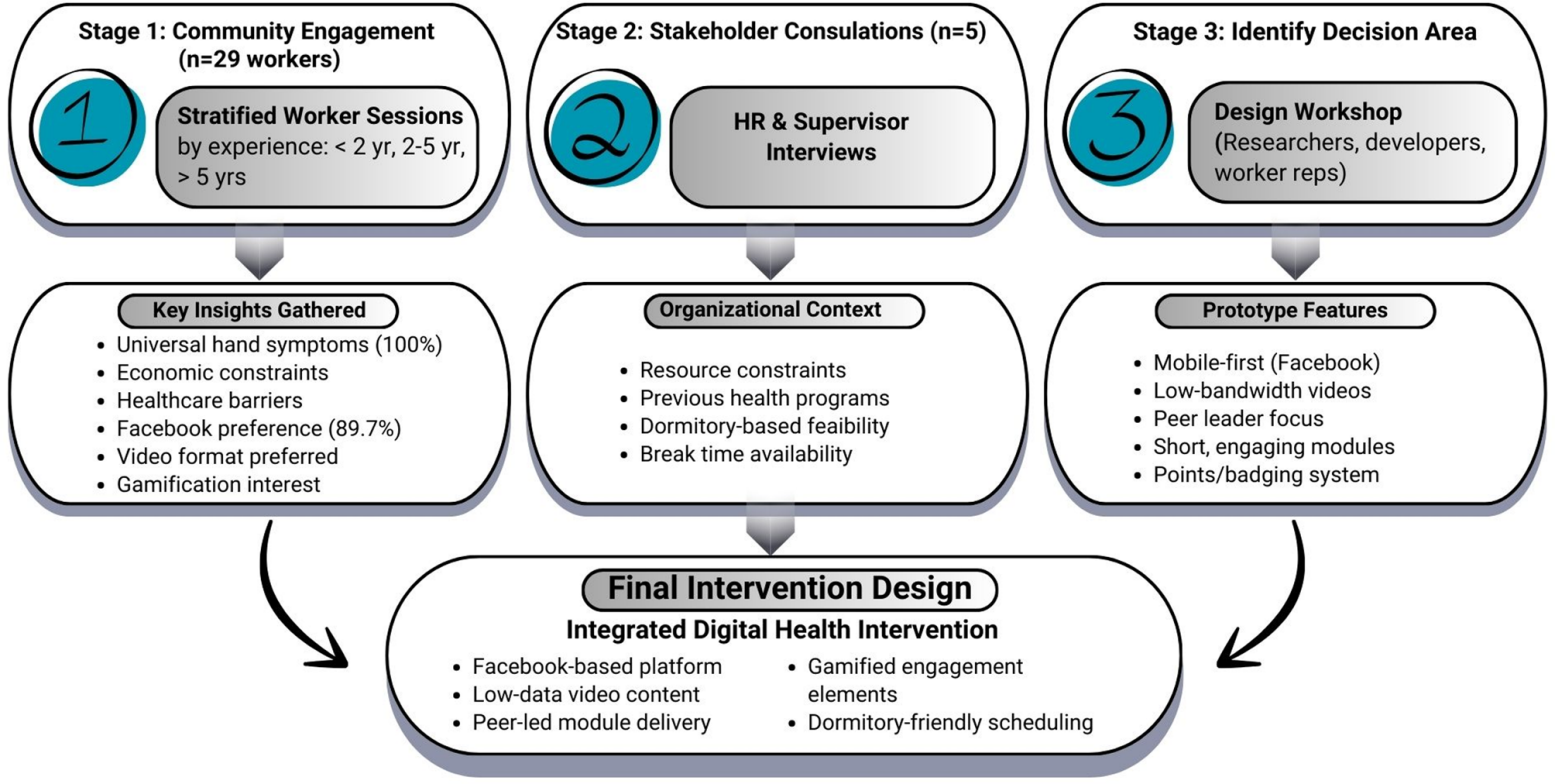
Co-design process- how engagement insights informed intervention development

Expert validation confirmed that the co-design process produced an intervention meeting rigorous clinical standards (S-CVI/Ave: 0.94) while maintaining fidelity to worker preferences and organizational constraints. Figure 2 illustrates how insights from all three-stage engagement process systematically informed each design decision, illustrating one approach through which community involvement may contribute interventions that balance community priorities with clinical rigor and organizational feasibility. The validated intervention now advances to Phase II implementation research for effectiveness evaluation.

## Discussion

### Principal insights

This paper demonstrates that engagement with Myanmar migrant workers produced a culturally appropriate, clinically validated digital health intervention for work-related musculoskeletal disorders. Three key insights emerged from the co-design process.

First, musculoskeletal symptoms exist across all experience levels. All 29 participating workers reported hand symptoms (numbness, tingling, pain), with most experiencing multiple complaints including neck pain (79%), lower back pain (72%), and ankle pain (45%) from repetitive seafood processing work.

Second, workers possess clear preferences shaped by structural constraints. Workers identified Facebook as their preferred delivery platform (89.7% daily use), rejected mandatory workshops on their day off, specified exercises must be dormitory-performable without equipment in maximum 15–20 min, and refined gamification features to emphasize participation over performance to avoid discouraging those with limitations.

Third, despite originating from community preferences and practical constraints rather than clinical protocols, the intervention achieved excellent content validity (S-CVI/Ave:0.94) when assessed by international physical therapy experts. This suggests, at least in this context, that community involvement and clinical rigour need not be competing goals though whether the resulting intervention produces measurable health improvements will be determined in Phase II.

### Methodological contributions and limitations

#### Methodological contribution

This work demonstrates a replicable approach for developing interventions with vulnerable populations. The three-stage process; stratified worker engagement (by experience level), stakeholder consultation (organizational feasibility), and expert validation (clinical appropriateness) captured distinct perspectives that researcher alone could not have anticipated. The alignment between worker preferences (dormitory-based, equipment-free) and organizational constraints (no facility modifications, outside work hours) emerged through engagement rather than researcher prediction. Future replications of this approach would benefit from prospective documentation of engagement costs including researcher time, participant compensation, and translation services to support other occupational health practitioners in planning and budgeting for similar community-based work.

#### Methodological limitations

Convenience sampling through workplace announcements likely attracted workers with stronger health concerns; symptom prevalence reflects participant experiences rather than population estimates. Single-site design and focus on Myanmar migrants in Thailand’s seafood industry limit generalizability to other migrant groups, industries, or countries. The methodological approach offers a transferable framework, but specific content requires cultural adaptation. The co-design process itself carries inherent limitations: engagement sessions were conducted on workers’ only day off, which may have constrained the depth and candour of participation. Workers were not involved in data analysis or manuscript preparation, meaning interpretation remained researcher-driven despite the participation framing. The sample of 29 workers from a single facility cannot be assumed to represent the broader Myanmar migrant workforce, and the three stratified groups, while informative, may not have captured the full range of experience s across this population.

### Ethical tensions and reflections

Engagement with vulnerable populations requires acknowledging tensions. As researchers from Chulalongkorn University, we entered a workplace where workers faced economic vulnerability and uncertain immigration status. Although the principal investigator’s Myanmar and native Burmese fluency reduced linguistic and cultural barriers, our institutional position likely shaped what workers felt comfortable sharing. Despite repeated assurances that participation was voluntary and would not affect employment, workers may have felt constrained in expressing disagreement with our proposals or raising workplace concerns beyond our project scope.

Workers gave up portions of their only rest day for hour-long sessions, despite exhaustion from demanding work schedules. While we provided compensation, we asked tired people to work on their day off to help us develop research.

We addressed these tensions through careful recruitment avoiding supervisor referrals, conducting sessions in Burmese, providing compensation, and validating worker expertise. However, these measures mitigate rather than eliminate power differentials. Acknowledging these limitations honestly, rather than claiming partnership we did not fully achieve, represents our attempt to practice research ethics grounded in reality.

### Transferable design principles

Several insights from this study may apply in other contexts, though their transferability is bounded by the specific conditions that shaped them: a workforce with limited employment alternatives, high rates of digital platform use, strong informal peer networks, and institutional constraints preventing facility modification. Cultural adaptation must extend beyond language translation to encompass values, imagery, and social structures. For Myanmar workers, this meant incorporating community support values and structuring peer challenges to emphasize participation over competition.

Digital delivery through familiar platforms can overcome multiple barriers simultaneously. Facebook addressed time constraints, privacy concerns, and linguistic barriers while enabling peer support. Gamification elements leverage social dynamics when designed collaboratively with workers refined proposed features to fit community values rather than accepting researcher-imposed designs.

Progressive exercise structures (mobility exercises for immediate relief, stretching for restricted movement, strengthening for prevention) accommodated varying symptom severity while remaining implementable without equipment in small spaces. The alignment between worker preferences and organizational constraints demonstrates how community involvement identifies feasible solutions researchers might overlook.

### Implications for migrant health research

The findings illuminate structural challenges facing migrant workers globally: economic pressures overriding health considerations, healthcare discrimination and barriers, and acceptance of health risks as economic trade-offs [9, 25]. Workers described understanding health consequences but feeling unable to reduce hours or seek adequate care given family financial obligations.

Healthcare barriers extend beyond language to discrimination and institutional exclusion. Workers reported disrespectful interpreters and noted workplace insurance covered only acute injuries while chronic conditions required payment from limited savings. Digital health approaches may therefore offer advantages by reaching workers outside healthcare systems that systematically fail them, leveraging platforms where migrants already communicate and maintain community connections.

However, individual interventions cannot fully address inequities rooted in structural conditions. Workers develop disorders from repetitive tasks and inadequate ergonomic design which are the conditions we could not change. While exercises provide symptom relief, they place health responsibility on workers rather than addressing employer obligations for safe conditions. Future research must grapple with this tension between achievable individual interventions and needed structural reforms. In particular, future studies should explore the integration of co-designed individual exercise programmes with employer-led ergonomic modification including workstation redesign, scheduled rest breaks, and task rotation to provide a more complete occupational health response than individual-level approaches alone can offer.

## Conclusions

Workers possess essential knowledge about their needs, constraints, and what works in their circumstances. When research incorporates worker input in intervention design, the result is content that is both clinically appropriate and practically feasible. It should be noted, however, the clinical validity as established through expert review does not confirm effectiveness; whether the intervention procedures measurable health benefits for the population remains to be determined in Phase II. However, genuine partnership with vulnerable populations faces inherent tensions: power differentials persist despite mitigation efforts, time poverty limits engagement depth, and individual interventions cannot resolve structural workplace conditions causing injuries.

The validated intervention advances to Phase II implementation research for effectiveness evaluation. The methodological framework described here stratified engagement, multi-stakeholder consultation, expert validation offers one approach, derived from a specific cultural and occupational context, for developing interventions with marginalised populations. Whether it transfers usefully to other settings, and whether the resulting intervention improves health outcomes, are questions that remain open and will be examined in subsequent research.

## Data Availability

The qualitative datasets generated during community engagement sessions contain sensitive information about a vulnerable migrant worker population and cannot be made publicly available to protect participant confidentiality and privacy. Anonymized thematic analysis summaries and expert validation data supporting the findings are available from the corresponding author upon reasonable request and subject to ethical approval.

## Declarations

### Authors’ information

PHH is a physical therapist and postdoctoral fellow specializing in occupational health and community-based participatory research with migrant populations. PYC is a Wellcome-funded researcher with extensive experience in ethics, community engagement and involvement in Southeast Asian contexts. STM is a public health specialist focusing on migrant health and occupational health interventions. SB is a communication scholar specializing in health communication innovation and community development approaches. PHH and STM are native Burmese speakers.

### Reporting guidelines

This manuscript was prepared in accordance with the GRIPP2 reporting guidelines for patient and public involvement and engagement in research [14]. The completed checklist is provided as a supplementary file and is available via the Open Science Framework (https://www.osf.io/wdg3c/files/tzn65).
